# Supplementary figures and images for: Genome-wide prediction of topoisomerase IIβ binding by architectural factors and chromatin accessibility
Source: PLoS Comput Biol. 2021 Jan 19;17(1):e1007814. doi: 10.1371/journal.pcbi.1007814 (PMC7845959; doi:10.1371/journal.pcbi.1007814)

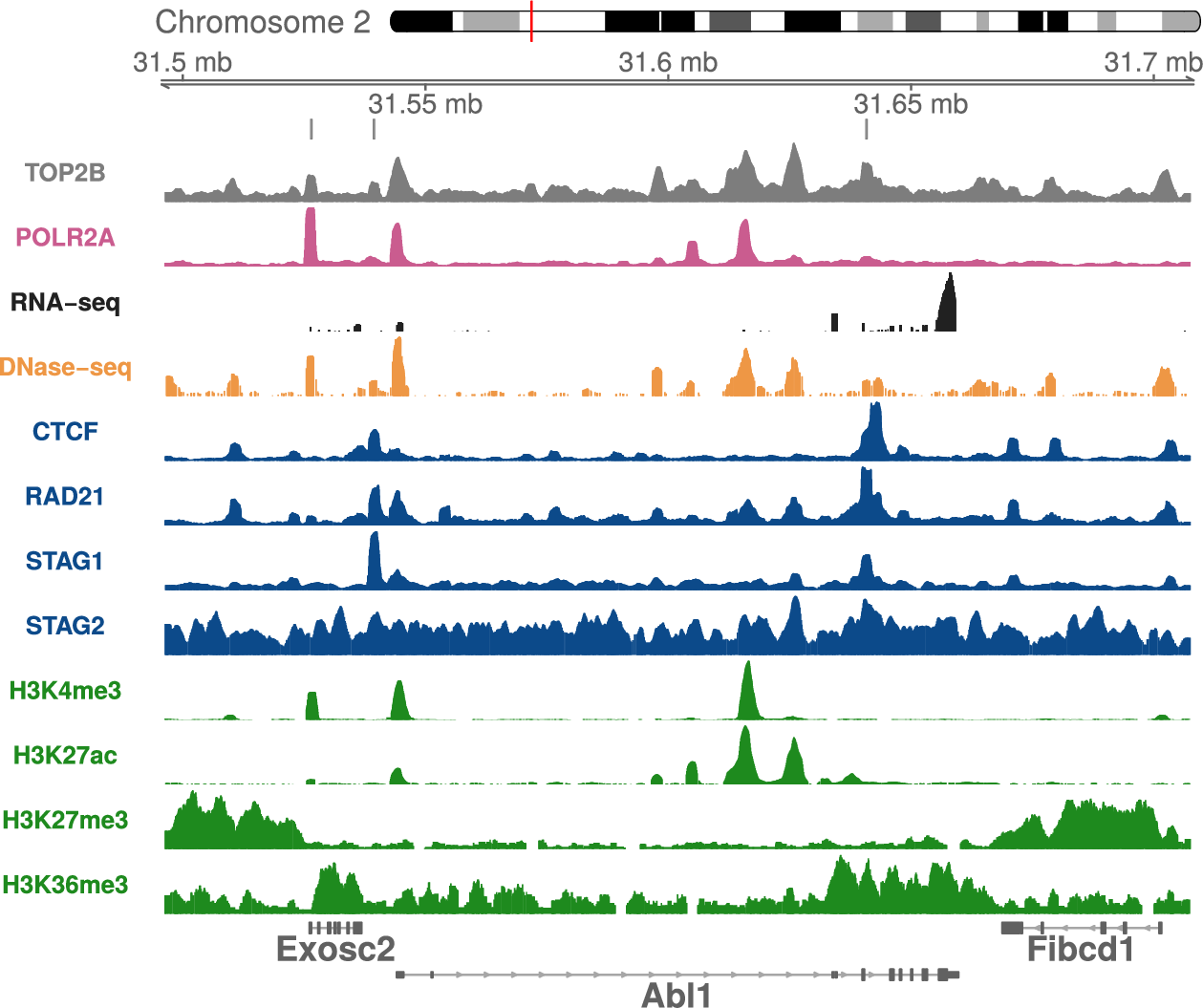

Supplement: S1 Fig — From top to bottom, TOP2B (grey), Pol2 (purple), RNA-seq (black), DNase-seq (yellow), architectural factors (blue) and a selection of histone marks (green) are displayed. (TIF) [file pcbi.1007814.s001.tif]

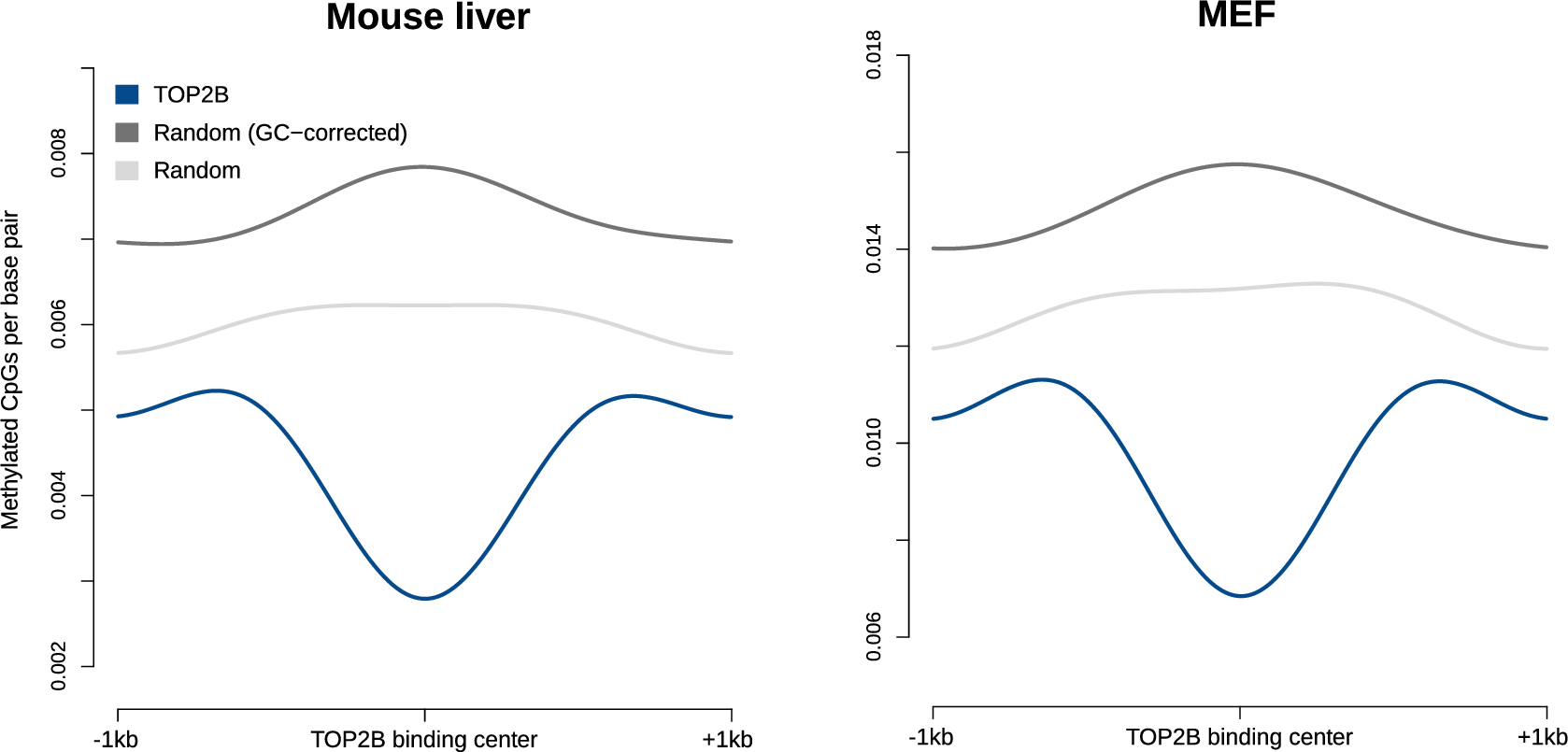

Supplement: S2 Fig — Average of whole genome bisulfite sequencing reads within ±1 kb of TOP2B binding center, random regions and GC corrected random regions are displayed. (TIF) [file pcbi.1007814.s002.tif]

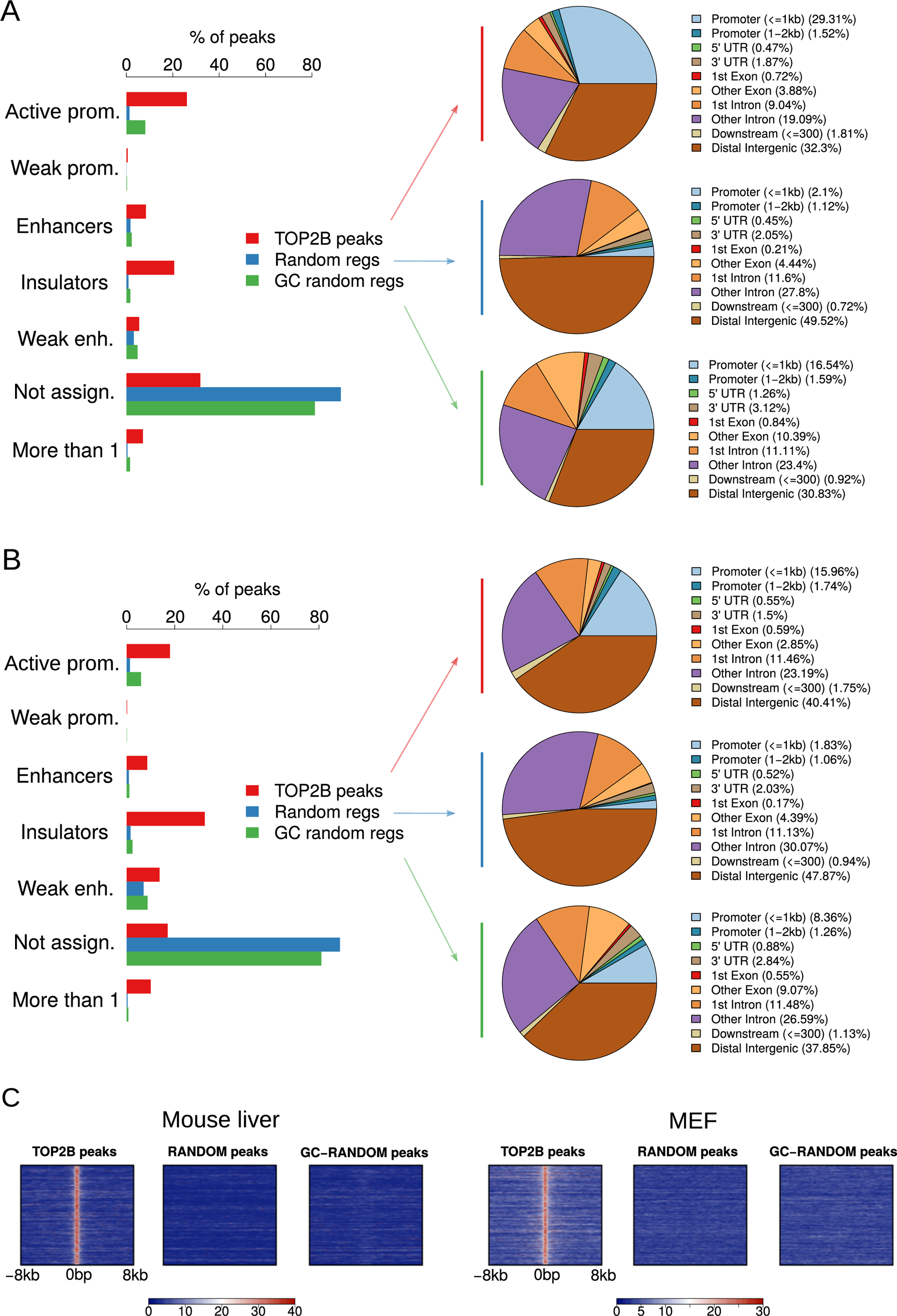

Supplement: S3 Fig — A. In the left panel, peaks were grouped into five classes as described in Matthews and Waxman, 2018 (see Materials and methods): promoters, weak promoters, enhancers, weak enhancers and insulators. The right panel displays pie charts showing peaks distribution relative to mouse TSSs. Data correspond to mouse liver.B. Same as (A) for MEFs data. C. Heatmap representations of TOP2B ChIP-seq reads enrichment within ± 8 kb of training peaks. (TIF) [file pcbi.1007814.s003.tif]

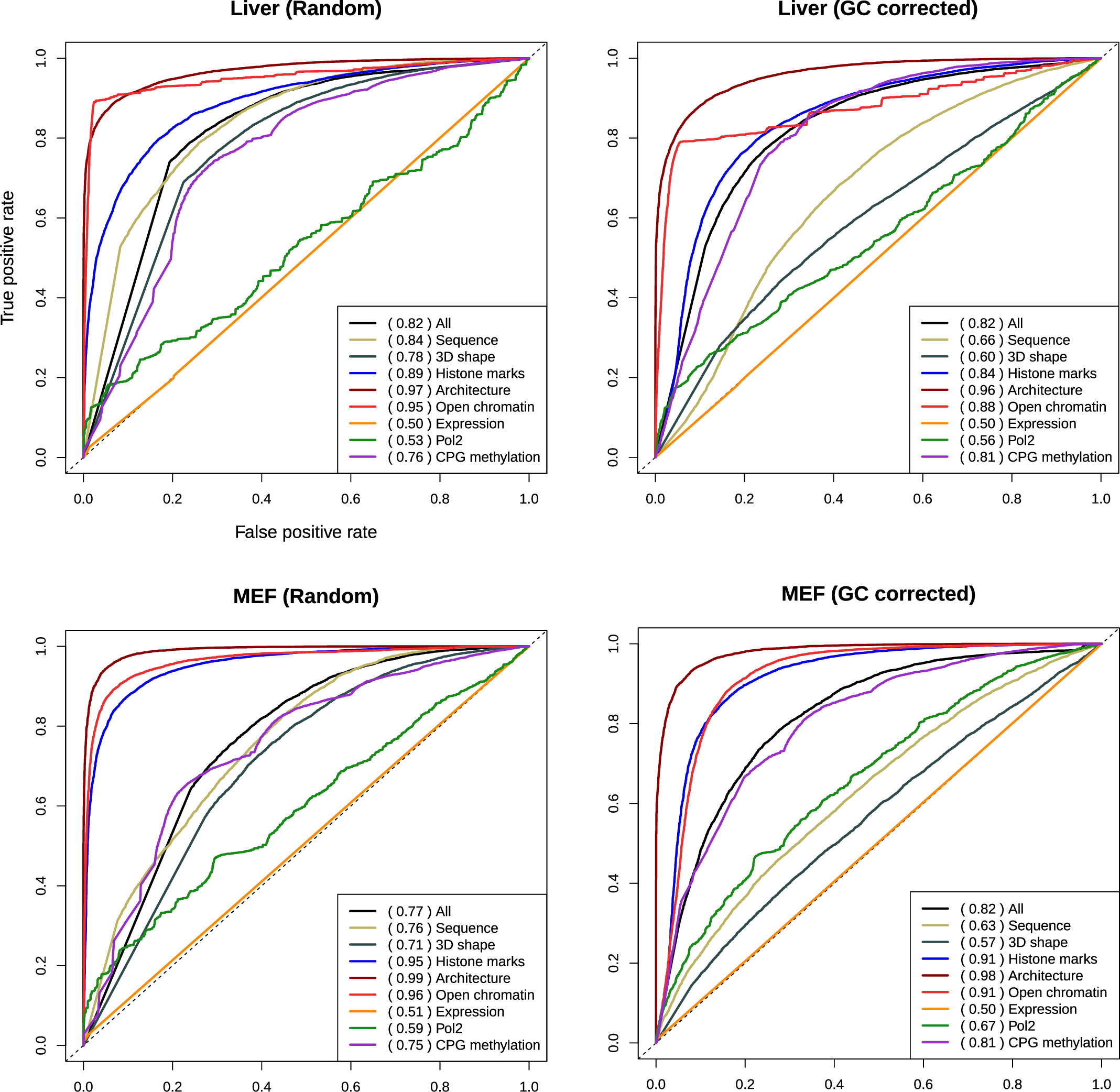

Supplement: S4 Fig — ROC curves and AUC values for Naive Bayes models trained on the indicated sets of features. (TIF) [file pcbi.1007814.s004.tif]

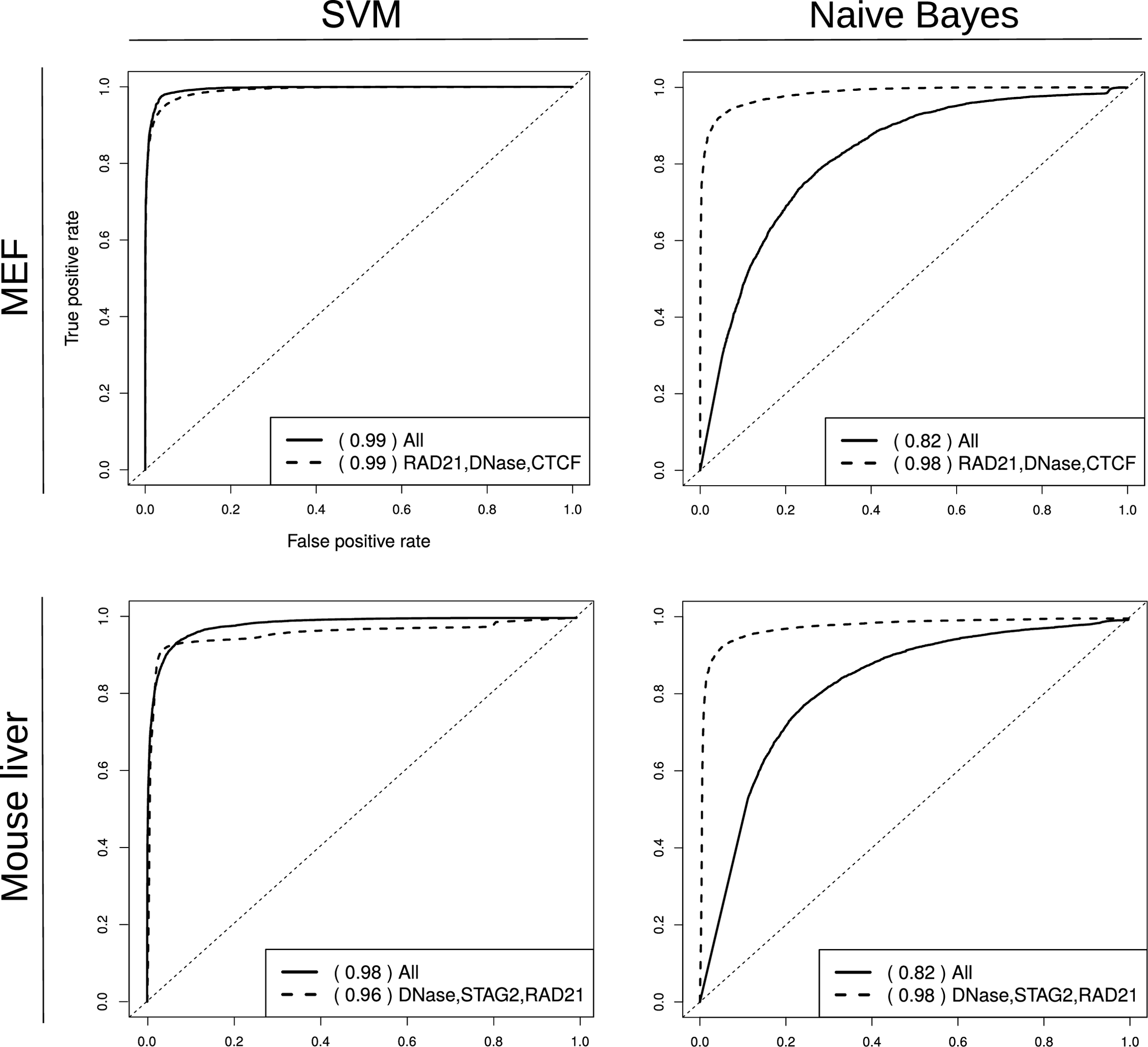

Supplement: S5 Fig — Models trained with a selection of 3 features show similar or even better performance than models trained with the whole set of features. (TIF) [file pcbi.1007814.s005.tif]

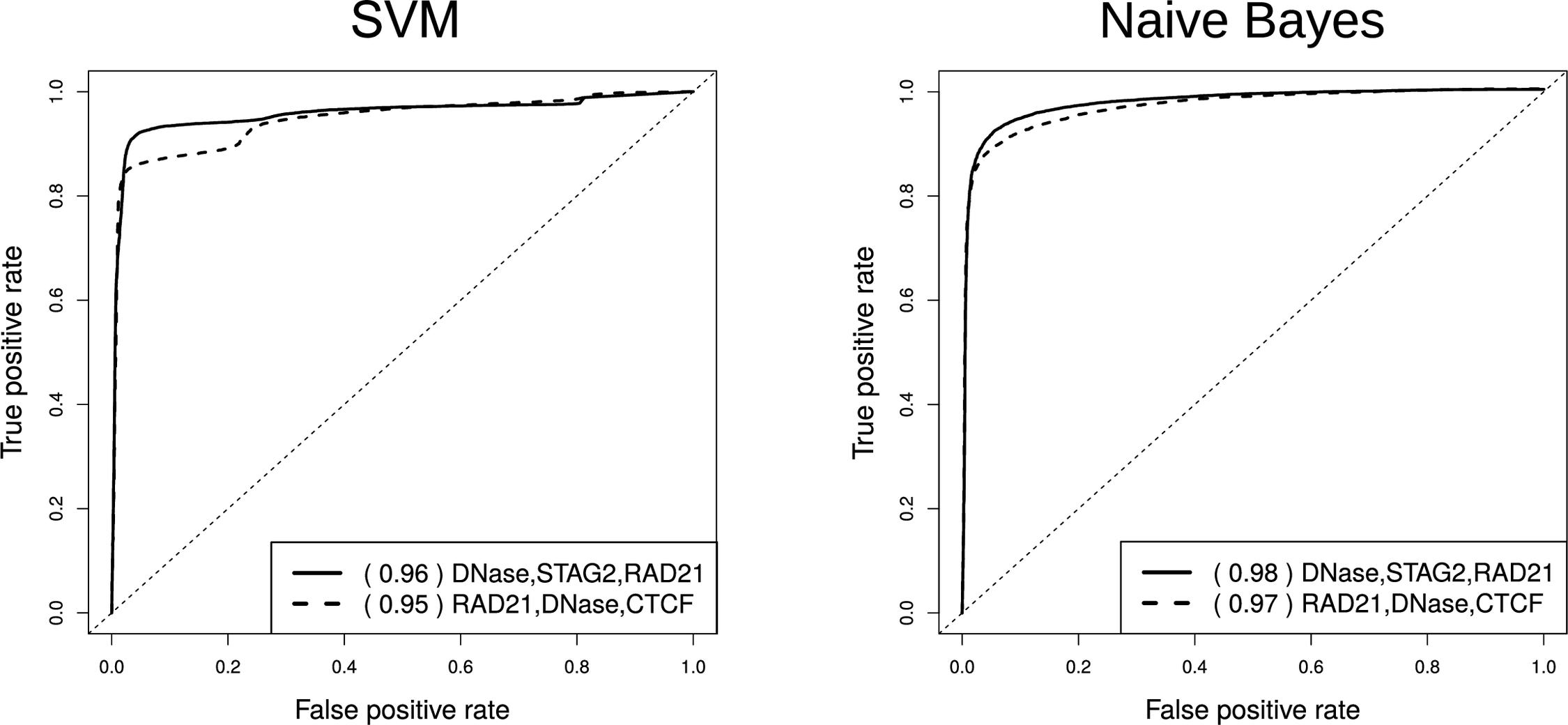

Supplement: S6 Fig — Models trained with either set of chromatin features show similar performances. (TIF) [file pcbi.1007814.s006.tif]

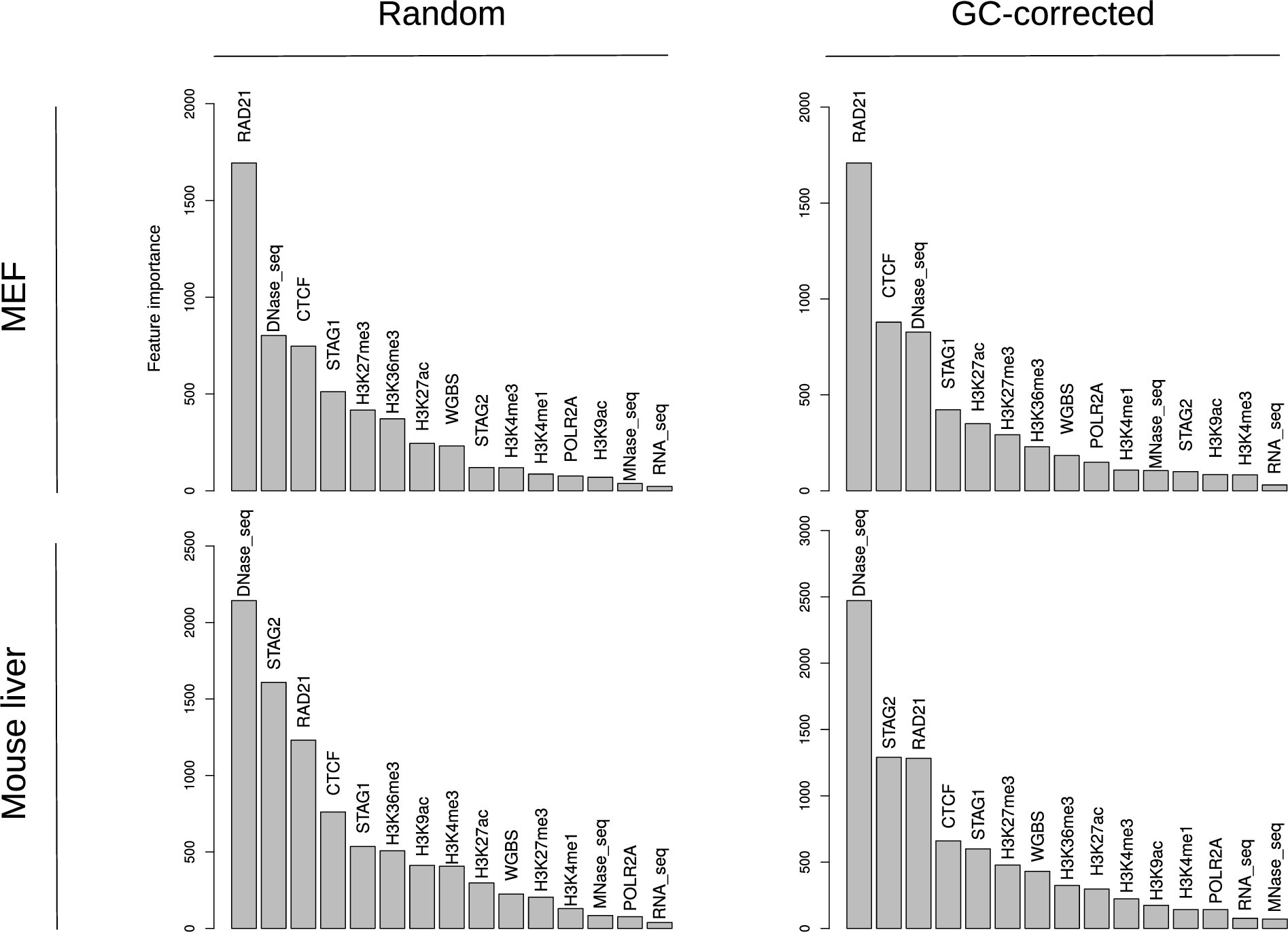

Supplement: S7 Fig — Only high-throughput sequencing datasets were considered. The predictive ability of the chromatin features is consistent with the feature selection analysis using Scatter Search and Fast Correlation-Based Filter. (TIF) [file pcbi.1007814.s007.tif]

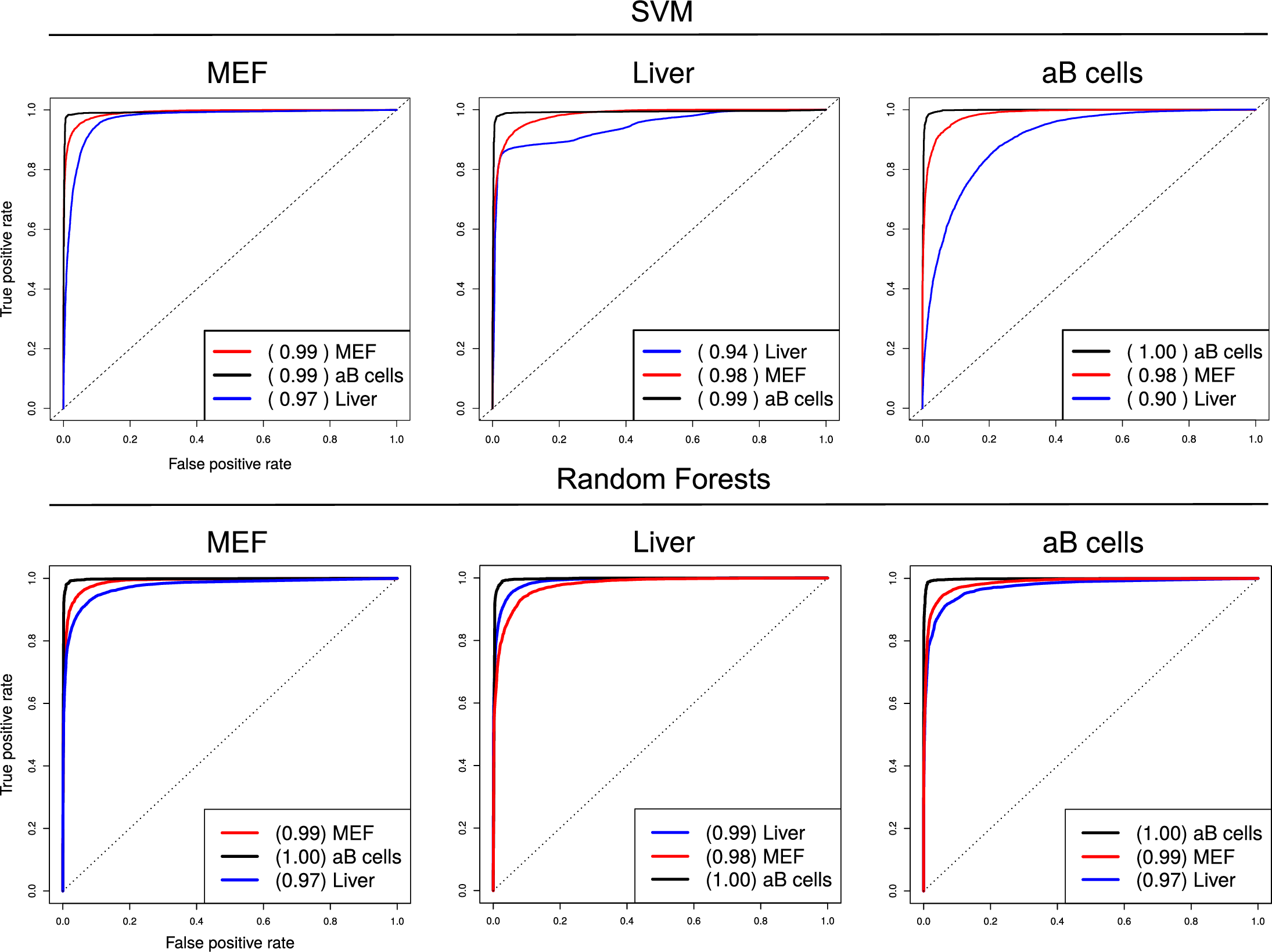

Supplement: S8 Fig — Models were trained on either MEF, liver or activated B cells and applied to the three systems. Only DNase-seq and ChIP-seq of RAD21 and CTCF were used for training. (TIF) [file pcbi.1007814.s008.tif]

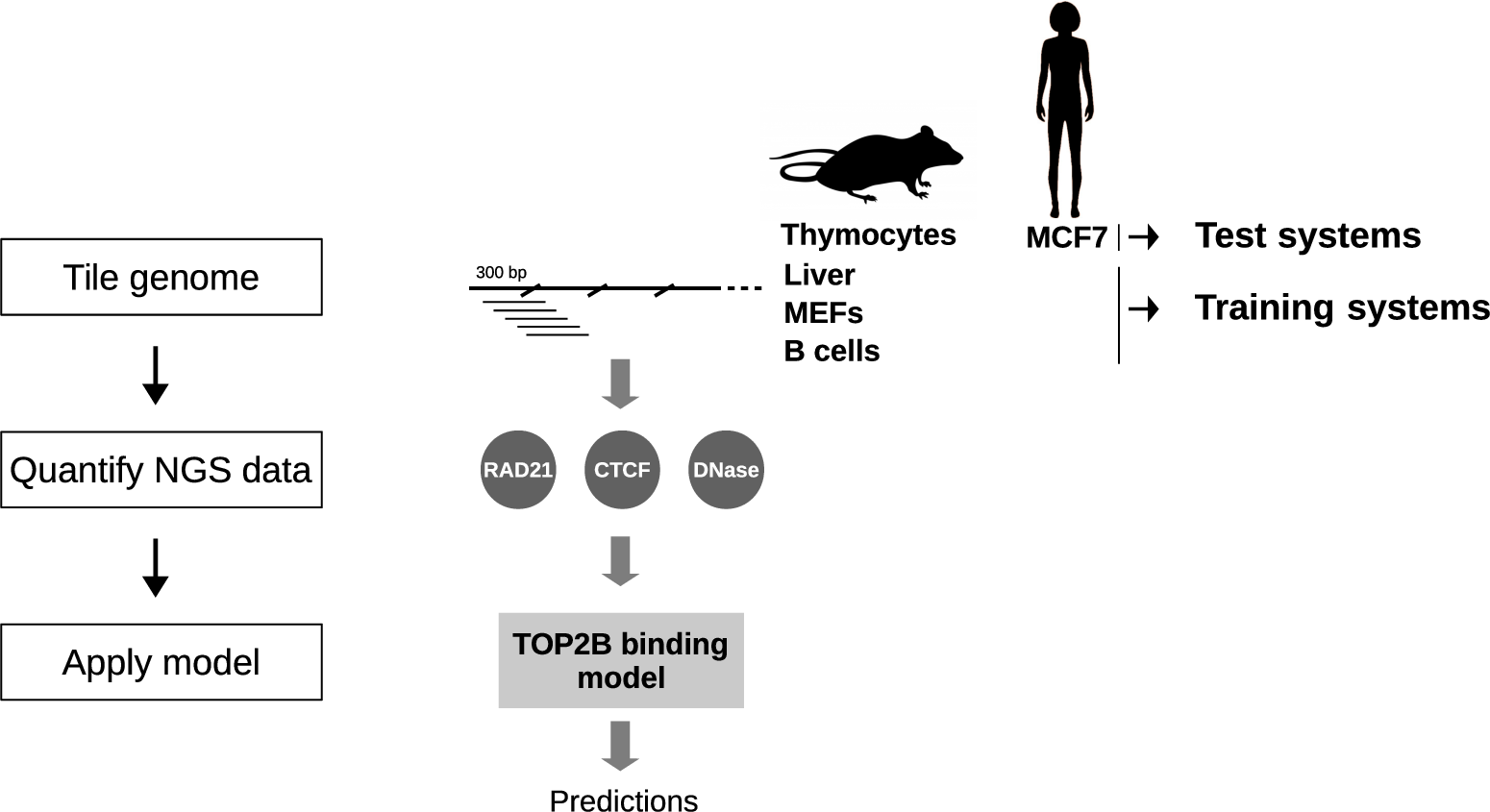

Supplement: S9 Fig — First, the genomes of mouse and human were tiled into bins of 300 bp with sliding windows of 50 bp. Then, DNase-seq and ChIP-seq reads of CTCF and RAD21 were scored on those bins (see Materials and methods) and the TOP2B binding model trained on mouse liver, MEFs and activated B cells was applied. Bins having a TOP2B probability higher than 0.95 were classified as TOP2B binding regions. Validation was performed on thymocytes and MCF7 cells for mouse and human, respectively. (TIF) [file pcbi.1007814.s009.tif]

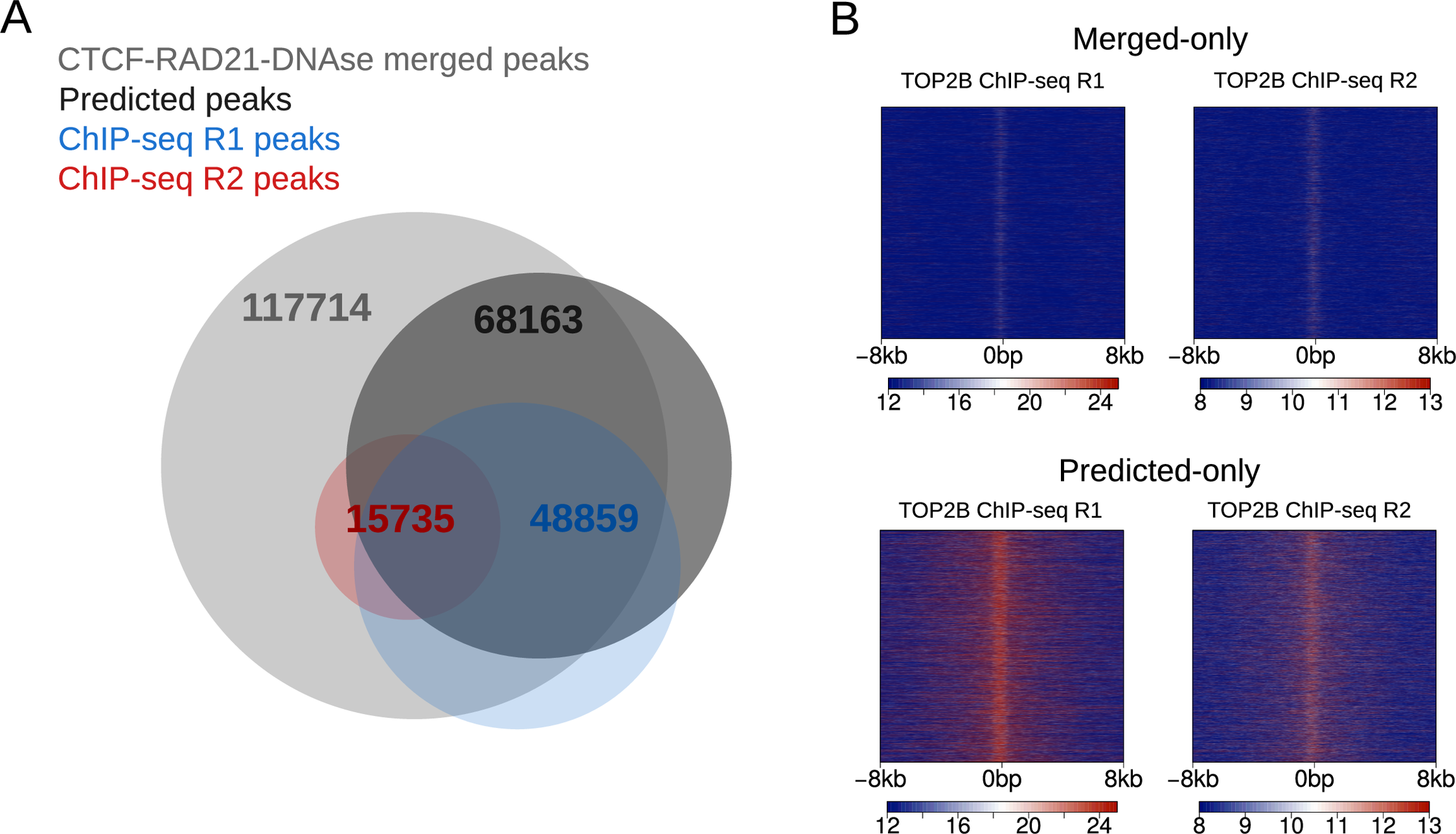

Supplement: S10 Fig — A. Venn diagram showing the overlaps between predicted TOP2B peaks (dark grey), DNase-CTCF-RAD21 peaks (light grey) and two replicates of experimental peaks (blue and red). B. Heatmap representations of TOP2B ChIP-seq reads enrichment within ± 8 kb of specific DNase-CTCF-RAD21 merged peaks (not predicted and not detected by TOP2B ChIP-seq) and specific predicted peaks (not included in the DNase-CTCF-RAD21 set and not detected by TOP2B ChIP-seq). The enrichment of TOP2B signal in the latter confirms the high sensitivity of our predictions. Peak calling was performed using HOMER. For illustration purposes, the same number of randomly selected peaks is represented in the two heatmaps. (TIF) [file pcbi.1007814.s010.tif]

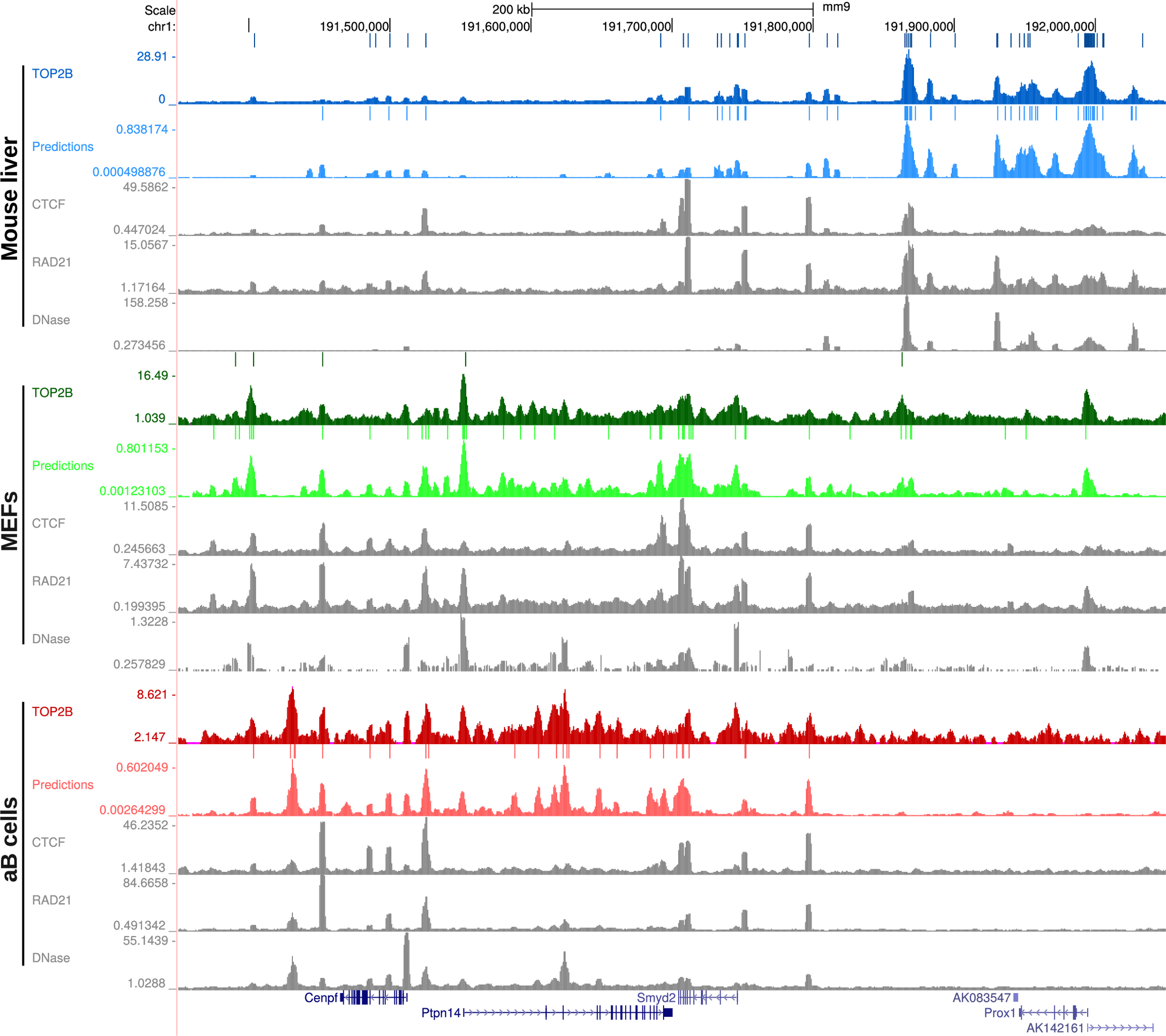

Supplement: S11 Fig — From top to bottom, genome browser view of TOP2B ChIP-seq, TOP2B virtual track, CTCF ChIP-seq, RAD21 ChIP-seq and DNase-seq are displayed for each system. TOP2B predicted and ChIP-seq peaks are also shown. (TIF) [file pcbi.1007814.s011.tif]

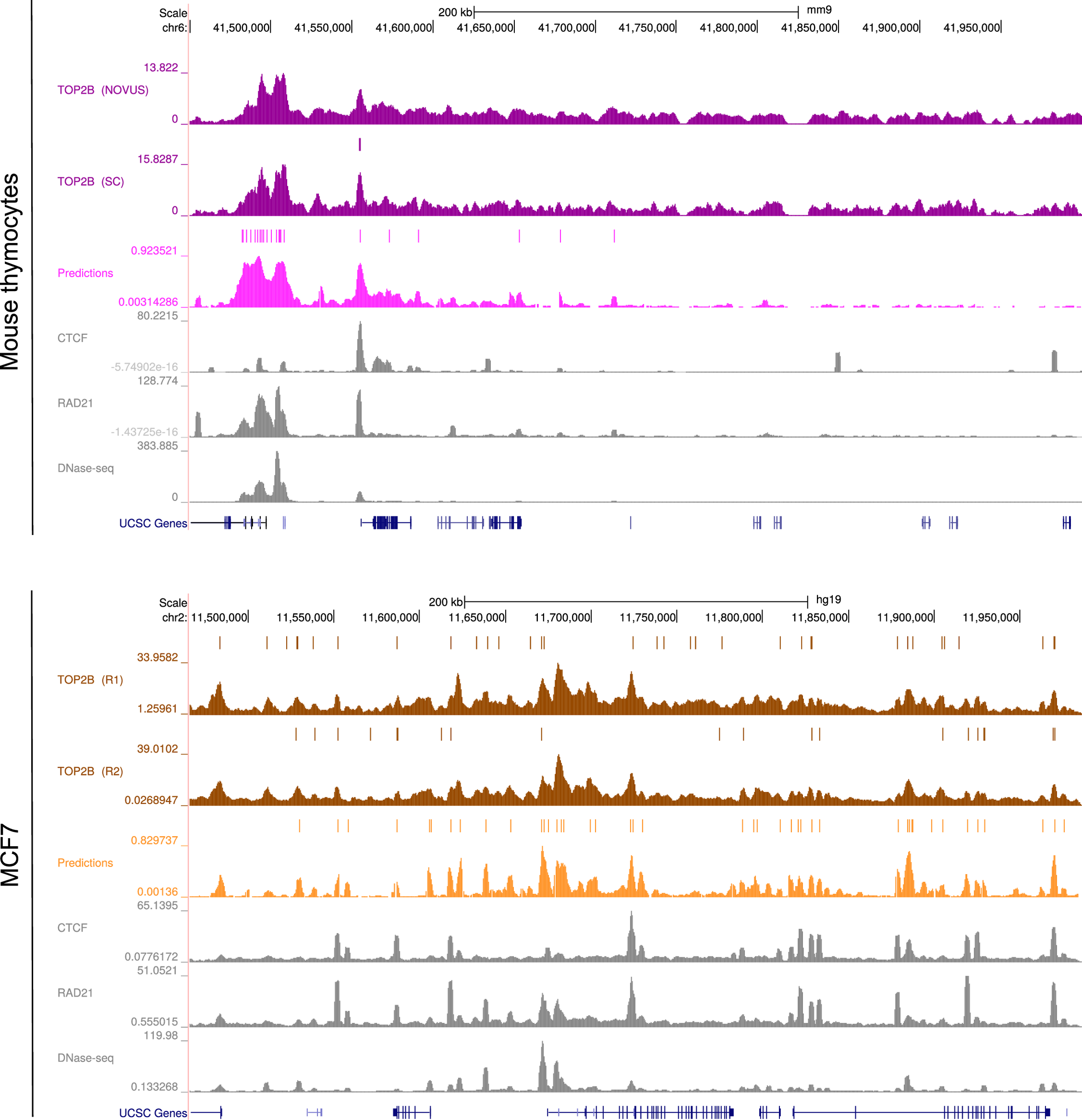

Supplement: S12 Fig — From top to bottom, genome browser view for TOP2B ChIP-seq, TOP2B predicted track, CTCF ChIP-seq, RAD21 ChIP-seq and DNase-seq. TOP2B predicted and ChIP-seq peaks are also shown. (TIF) [file pcbi.1007814.s012.tif]

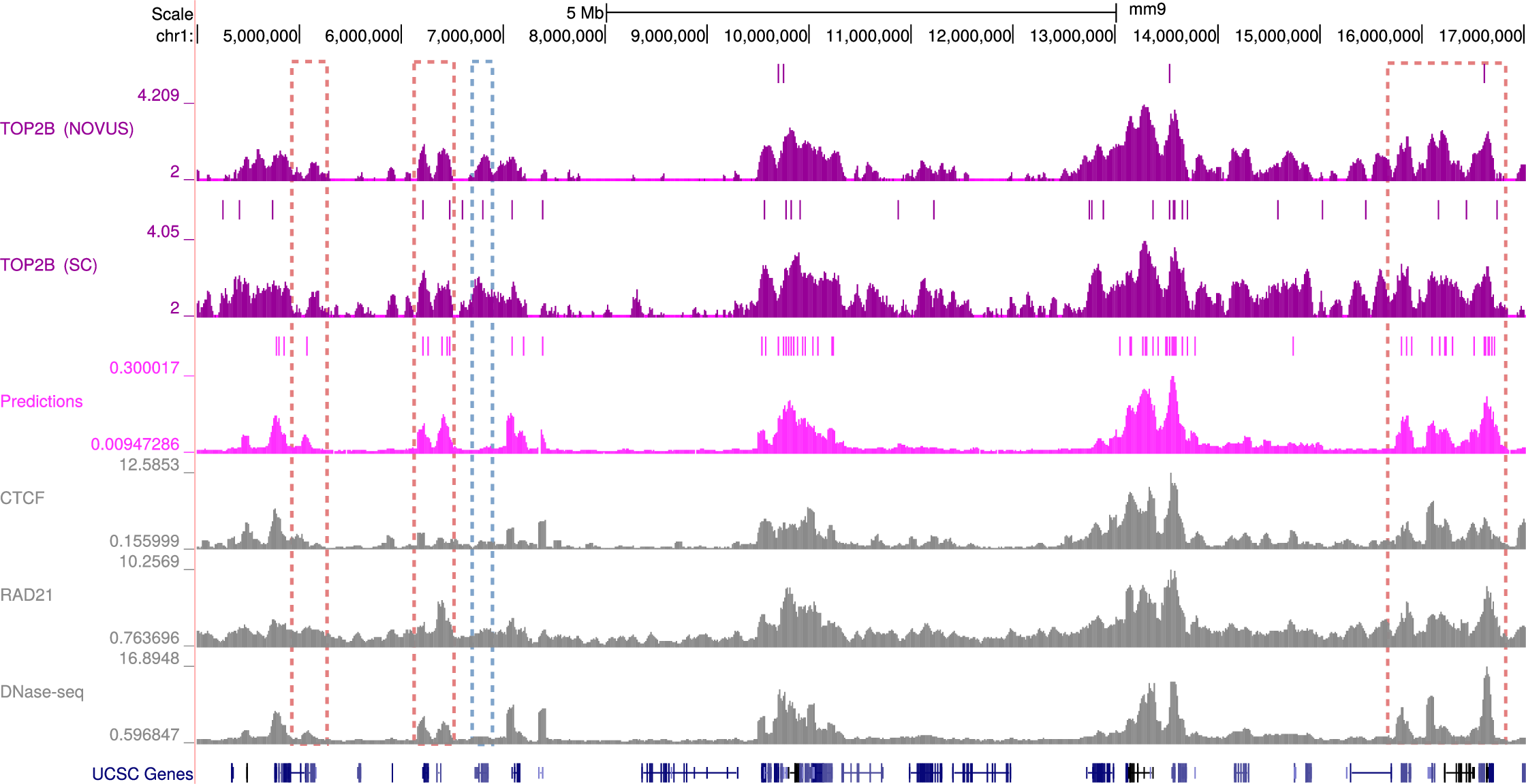

Supplement: S13 Fig — From top to bottom, genome browser view for TOP2B ChIP-seq using Novus and Santa Cruz antibodies, TOP2B predicted track, CTCF ChIP-seq, RAD21 ChIP-seq and DNase-seq. A. Highlighted in red are examples of true TOP2B binding sites only detected by our predictive approach. An example of one TOP2B binding site only detected by Santa Cruz antibody is highlighted in blue. Our TOP2B predictive signal displays an increased probability at such region, although lower than the threshold of 0.95. (TIF) [file pcbi.1007814.s013.tif]

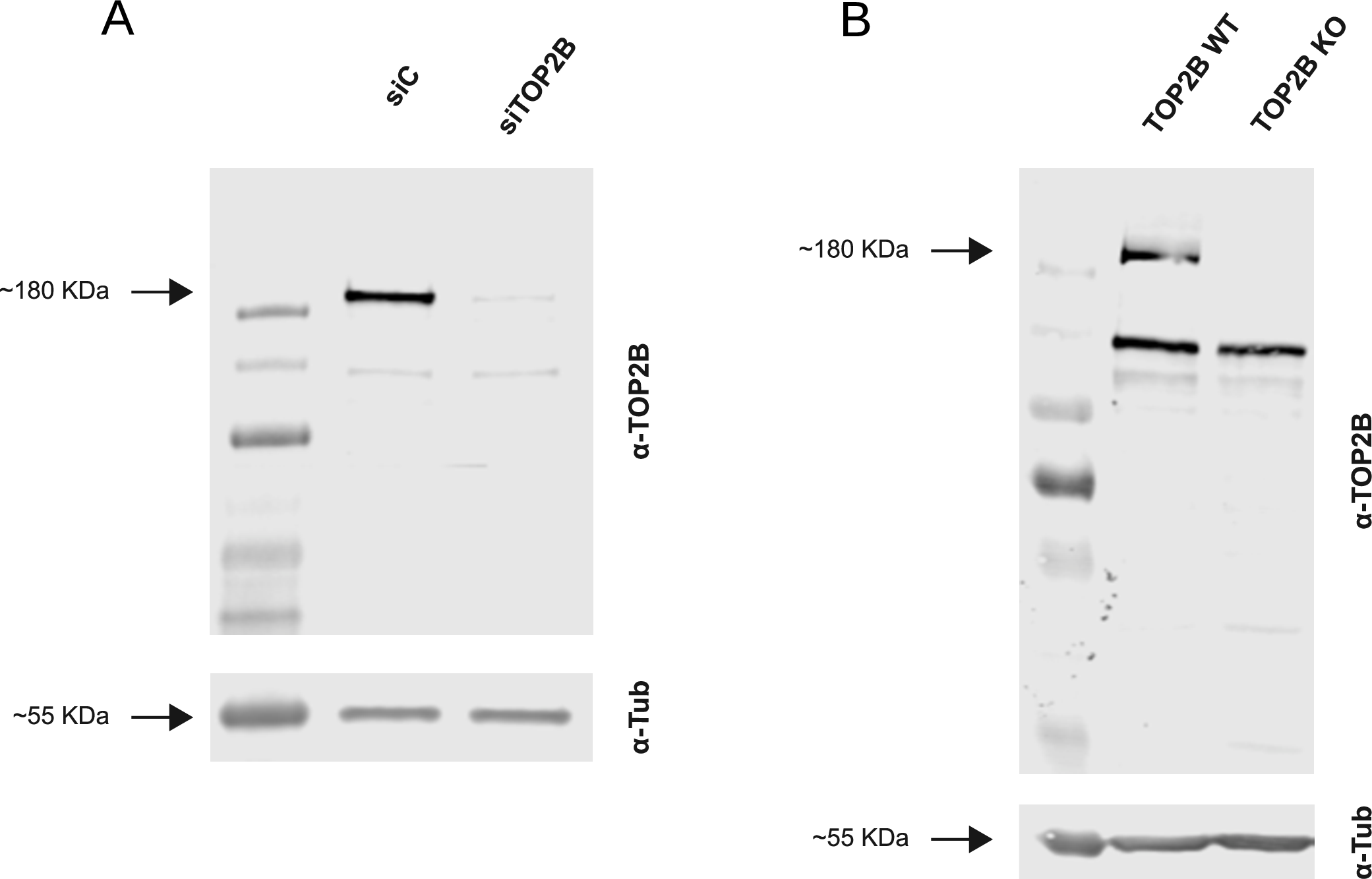

Supplement: S14 Fig — A. Western-blot of total MCF7 cell extract depleted (siTOP2B) or not (siC) for TOP2B with antibodies against TOP2B or α-Tubulin as a loading control. B. As in (A) with extracts from wild type (WT) and TOP2B-knock out (KO) MEFs. (TIF) [file pcbi.1007814.s014.tif]

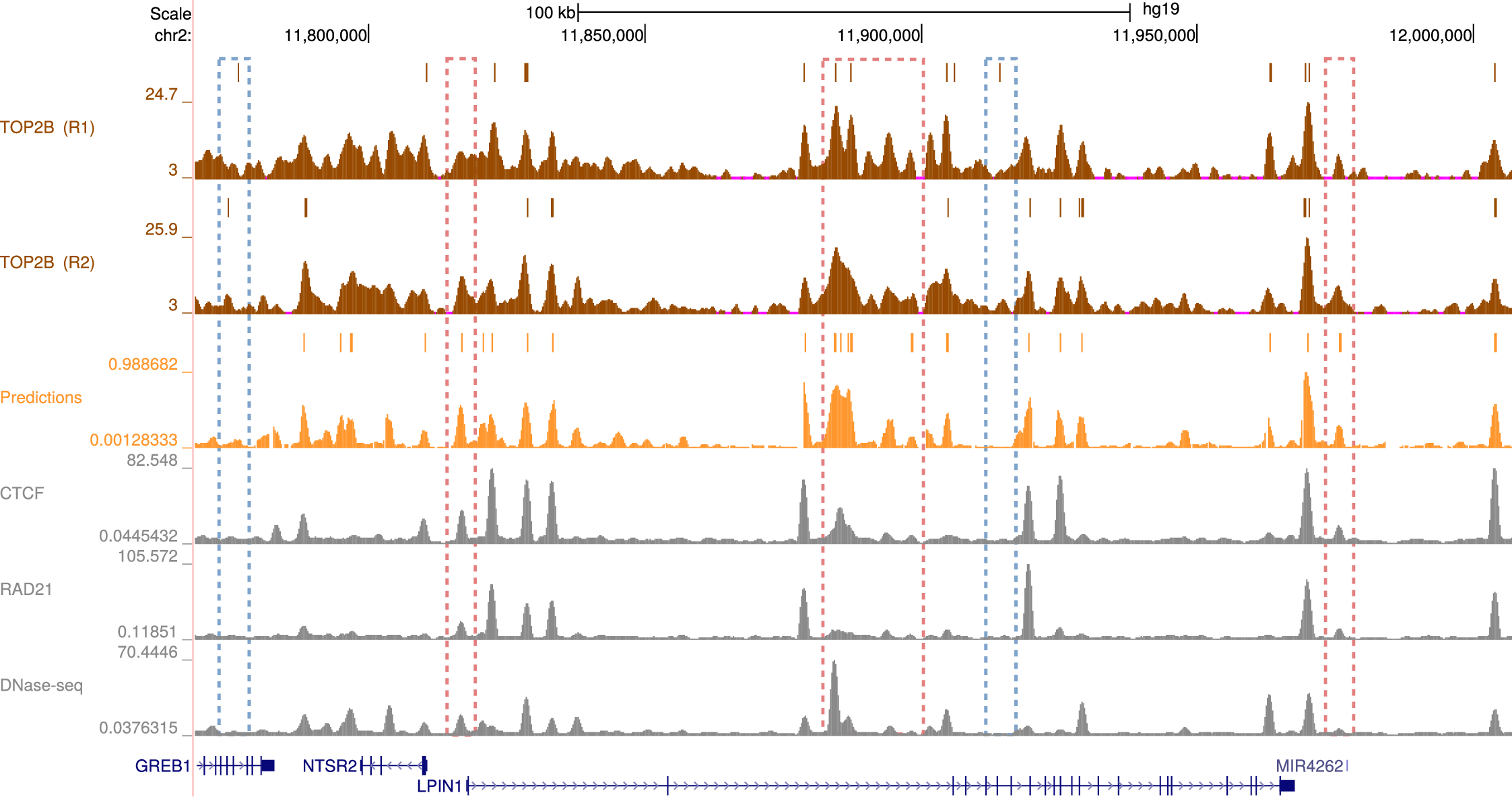

Supplement: S15 Fig — From top to bottom, genome browser view for TOP2B ChIP-seq, TOP2B predicted track, CTCF ChIP-seq, RAD21 ChIP-seq and DNase-seq. Examples of true TOP2B binding sites only detected by our predictive approach are highlighted in red and examples of TOP2B binding sites only detected by either ChIP-seq sample replicate are highlighted in blue. Our TOP2B predictive signal displays an increased probability at such regions, although lower than the threshold of 0.95. (TIF) [file pcbi.1007814.s015.tif]

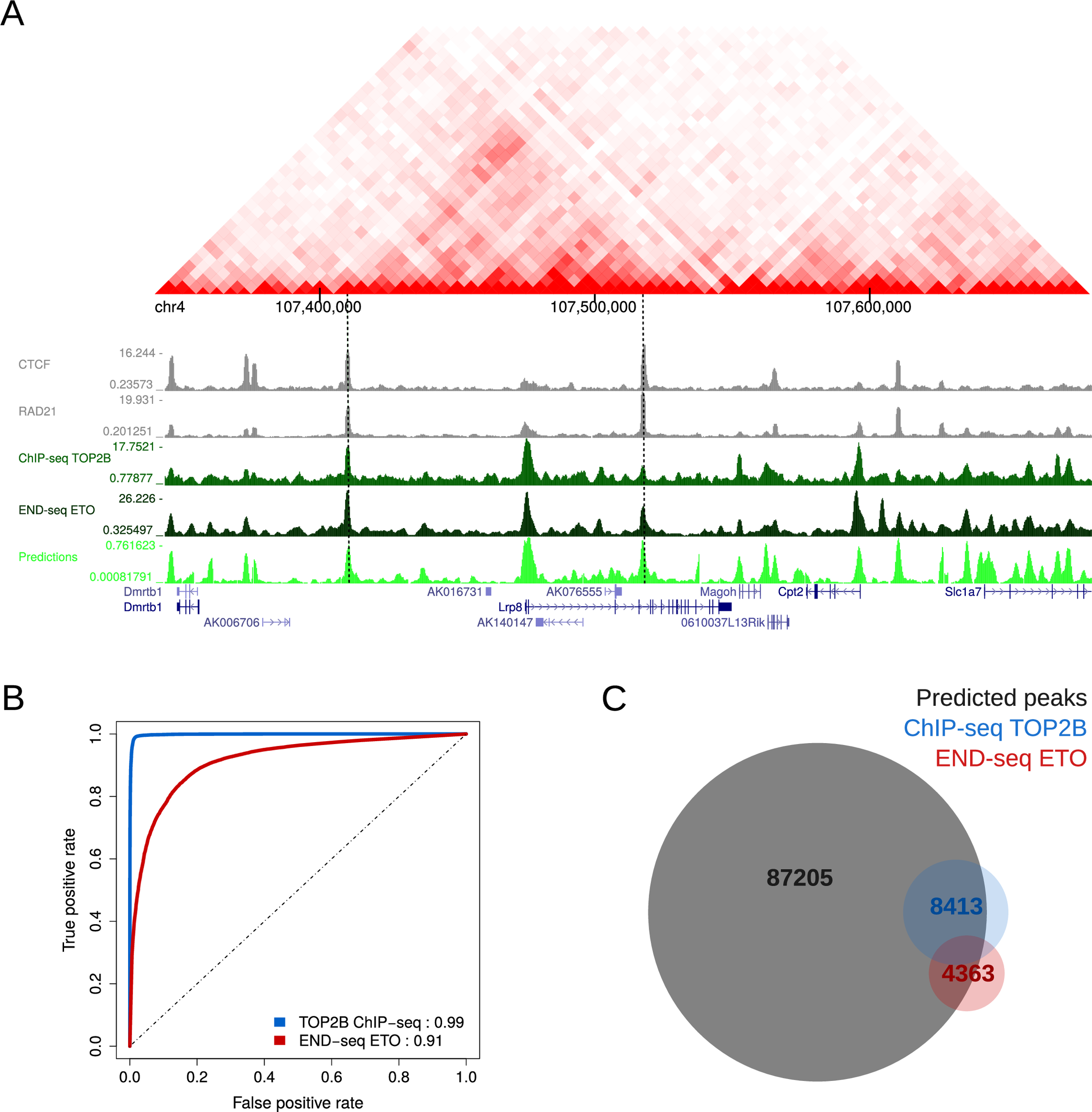

Supplement: S16 Fig — A. Genome browser view illustrating TOP2B activity at loop anchors. From top to bottom, Hi-C contact enrichment, CTCF, RAD21 and TOP2B occupancy measured by ChIP-seq, DSBs enrichment measured by END-seq and TOP2B predicted track. The left and right anchors of a loop near the Lrp8 gene are highlighted by dark grey dashed lines. B. ROC curves and AUC values for the prediction of END-seq and ChIP-seq peaks using our generalizing model. C. Venn diagram showing the overlaps between predicted TOP2B peaks (dark grey), END-seq peaks (red) and ChIP-seq peaks (blue). Peak calling was performed using MACS2. (TIF) [file pcbi.1007814.s016.tif]
