# Supplementary material for: Genome-wide prediction of topoisomerase IIβ binding by architectural factors and chromatin accessibility
Source: PLoS Comput Biol. 2021 Jan 19;17(1):e1007814. doi: 10.1371/journal.pcbi.1007814 (PMC7845959; doi:10.1371/journal.pcbi.1007814)
Supplement: S3 Table — (DOC) [file pcbi.1007814.s019.doc]

| **Selected Features** | **Accuracy** | |
| --- | --- | --- |
|  | Support Vector Machines | Naive Bayes |
| DNase-RAD21-CTCF | 0.901 ± 0.0363 | 0.916 ± 0.0042 |
| DNase-RAD21-STAG2 | 0.932 ± 0.0170 | 0.929 ± 0.0048 |

**S3 Table.** Performance of SVM and NB classifiers trained either with DNase, RAD21 and CTCF or DNase, RAD21 and STAG2 in mouse liver using GC-corrected background regions.
