# Supplementary material for: Genome-wide prediction of topoisomerase IIβ binding by architectural factors and chromatin accessibility
Source: PLoS Comput Biol. 2021 Jan 19;17(1):e1007814. doi: 10.1371/journal.pcbi.1007814 (PMC7845959; doi:10.1371/journal.pcbi.1007814)
Supplement: S4 Table — Percentage of correctly classified classes are shown. (DOC) [file pcbi.1007814.s020.doc]

| **System** | **Random** | | **GC-corrected** | |
| --- | --- | --- | --- | --- |
|  | TOP2B | NON-TOP2B | TOP2B | NON-TOP2B |
| Mouse liver | 0.97 | 0.98 | 0.95 | 0.96 |
| MEF | 0.99 | 0.98 | 0.95 | 0.97 |

**S4 Table.** Confusion matrix corresponding to Random Forests models trained with all chromatin features but DNA sequence and 3D DNA shape. Percentage of correctly classified classes are shown.
