# Supplementary material for: Genome-wide prediction of topoisomerase IIβ binding by architectural factors and chromatin accessibility
Source: PLoS Comput Biol. 2021 Jan 19;17(1):e1007814. doi: 10.1371/journal.pcbi.1007814 (PMC7845959; doi:10.1371/journal.pcbi.1007814)
Supplement: S5 Table — ChIP-seq reads and probabilities were log2-transformed before comparison. Signal correlations were then computed at experimental ChIP-seq peaks indicated in the first column. (DOC) [file pcbi.1007814.s021.doc]

| **Mouse liver** | ChIP-seq (R1) | ChIP-seq (R2) | Predictions |
| --- | --- | --- | --- |
| ChIP-seq (R1) | 1 | 0.78 | 0.65 |
| ChIP-seq (R2) | 0.81 | 1 | 0.68 |
| **MEFs** | ChIP-seq | | Predictions |
| ChIP-seq | 1 | | 0.51 |
| **aB** | ChIP-seq | | Predictions |
| ChIP-seq | 1 | | 0.45 |

**S5 Table.** Pearson’s correlation coefficients between experimental TOP2B ChIP-seq replicates and predictions in mouse liver, MEFs and activated B cells. ChIP-seq reads and probabilities were log2-transformed before comparison. Signal correlations were then computed at experimental ChIP-seq peaks indicated in the first column.
