# Supplementary material for: Genome-wide prediction of topoisomerase IIβ binding by architectural factors and chromatin accessibility
Source: PLoS Comput Biol. 2021 Jan 19;17(1):e1007814. doi: 10.1371/journal.pcbi.1007814 (PMC7845959; doi:10.1371/journal.pcbi.1007814)
Supplement: S7 Table — ChIP-seq reads and probabilities were log2-transformed before comparison. Signal correlations were then computed at experimental ChIP-seq peaks indicated in the first column. (DOC) [file pcbi.1007814.s023.doc]

|  | ChIP-seq (R1) | ChIP-seq (R2) | Predictions |
| --- | --- | --- | --- |
| ChIP-seq (R1) | 1 | 0.66 | 0.49 |
| ChIP-seq (R2) | 0.69 | 1 | 0.54 |

**S7 Table.** Pearson’s correlation coefficients between experimental TOP2B ChIP-seq replicates and predictions in MCF7. ChIP-seq reads and probabilities were log2-transformed before comparison. Signal correlations were then computed at experimental ChIP-seq peaks indicated in the first column.
