# Supplementary material for: Genome-wide prediction of topoisomerase IIβ binding by architectural factors and chromatin accessibility
Source: PLoS Comput Biol. 2021 Jan 19;17(1):e1007814. doi: 10.1371/journal.pcbi.1007814 (PMC7845959; doi:10.1371/journal.pcbi.1007814)
Supplement: Attachment — Submitted filename: revision_letter.pdf [file pcbi.1007814.s024.pdf]

Dear Editor,

We would like to thank you for the opportunity to submit a revised version of our manuscript. We would also like to express our gratitude to the reviewers for their feedback and helpful comments. We have revised the manuscript according to their suggestions, which we think has significantly improved its content. We sincerely hope that the manuscript is now suitable for publication.

Please find below our point-by-point response to specific reviewers' comments (note that line numbers refer to the manuscript version with tracked changes):

Reviewer #1

The authors utlise a number of publically available chip seq data sets for TOP2B and also for CTCF, RAD21, STAG1, STAG2, and a number of histone modifications. They use a range of computing methods to compare the genomic locations of TOP2B peaks/enriched regions with the peaks for the other types of proteins (CTCF, RAD21 etc). They also look at chromatin accessibility (DNAase hypersentivity) and various DNA features including CpG islands. They do find concordance between TOP2B peaks DNAase hypersenitive regions and CTCF and RAD21. However this is not surprising, as in ref 11 Uuskula-Reimand et al, they previously showed and reported in detail that half of CTCF and cohesin (contains RAD21) - part of abstract from Ref 11 copied below
"TOP2B associates with DNase I hypersensitivity sites, allele-specific transcription factor (TF) binding, and evolutionarily conserved TF binding sites on the mouse genome. Approximately half of all CTCF/cohesion-bound regions coincided with TOP2B binding. Base pair resolution ChIP-exo mapping of TOP2B, CTCF, and cohesin sites revealed a striking structural ordering of these proteins along the genome relative to the CTCF motif. These ordered TOP2B-CTCF-cohesin sites flank the boundaries of topologically associating domains (TADs) with TOP2B positioned externally and cohesin internally to the domain loop."
https://www.ncbi.nlm.nih.gov/pubmed/27582050

Although this reference is cited in the introduction of this study, this study fails to fully address how the findings in this manuscript relate to this previous closely related research. The authors should consider revising their Introduction and Discussion to reference more fully the closely related previous literature.

We have introduced several changes to fully address this question. On the one hand, we have extended some paragraphs from the "Introduction" and "Results and discussion" sections (lines 22-25, 44-45, 251). In addition, we have performed two new analyses that we included in "Results and Discussion" by adding a new paragraph (lines 512-529) and a new section ("Prediction of TOP2-induced DSBs", lines 658-679) where we specifically discuss our results in the context of Uusküla-Reimand et al (2016) and Canela et al (2017) findings.

This study also reports the generation two new datasets one from mouse cells and one from human cells to utlise their machine learning programmes to determine if they can predict where the TOP2B peaks/enriched regions will be found. In the venn diagrams in Figure 5B and 5E approx half the predicted TOP2B sites (based on the location of CTCF, RAD21 and DNAse hypersentive sites) overlap with the experimentally determined TOP2B sites. This agrees with the 50% concordance published in ref 11. What might be the reasons for the concordance not being 100%?

We would like to clarify that for this manuscript we have generated new datasets of TOP2B binding in MCF7 cells, the ones corresponding to mouse thymocytes are published (Álvarez-Quilón 2020). We have now clarified this further in the text (lines 555-556, 613-614). Regarding the partial (non-100%) concordance between our predicted and experimental peaks, we believe this reflects an

intrinsic limitation of peak calling, as opposed to other more quantitative analyses. Thus, even with two experimental replicates, with the same cell line and the same antibody, overlap between the called peaks is only 28-36 %. In our case, this is likely to be more accused, since we are converting a quantitative probability signal into a qualitative score by simply applying an arbitrary threshold of 0.95. As a matter of fact, as we show in Figures 6,S10,S13 and S15, predicted peaks not identified experimentally still harbor clear experimental TOP2B signal, despite not being selected by peak calling algorithms, and vice versa.

This manuscript confirms what was was previously reported for murine cells, using different computer programmes.

We agree that the main contributions of our study are not the biological discoveries regarding TOP2B binding themselves, but the development of machine learning models for the systematic generation of virtual TOP2B ChIP-seq tracks. We have now included several changes in the manuscript to made this aspect clearer.

Reviewer #2

In this manuscript Martinez-Garcia et al. develop a computational tool to predict the localization of TOPO2 beta genome wide based on input data sets from DNaseI, and Chip-Seq of CTCF and RAD21. The model can be used to predict both mouse and human TOPO2 beta localization. These results are based on the previously observed strong co-localization of TOPO2 beta with CTCF, cohesion and DNaseI accessible sites from several research groups in both mouse and human cells. The models show very good accuracy, with reported ~95% of TOPO2 beta peaks called using the trained sets with the models, and ~90% accuracy using the experimental data sets (MCF7, mouse thymocytes). Overall this is a well-done manuscript. However, given the strong co-localization of TOPO2 beta with the features used to develop the models (DNaseI, CTCF and cohesion), its utility is somewhat diminished. I recommended publication of this manuscript provided the following concerns are addressed.

Major Points

1.) It has been well known that TOPO2 beta co-localizes with DNaseI, CTCF and cohesion binding sites. It seems that much of the TOPO2 binding can be predicted summing the unique peak calls from the three chromatin elements (Fig 1C). What is the overlap frequency of TOPO2 beta peaks to the combined CTCF, DNaseI and Rad21 peaks (removing redundant peak calls – Bedtools Merge function)? Can this simple method achieve worse/similar/better accuracy that the computational methods? Can this analysis be added to Figure 5 (or as a supplement). A similar presentation can be used as in Figure 5 with the merged DNAseI, CTCF and Rad21 peak data set replacing the “Predicted” data set from the authors models. This could serve as good control as to the superiority of the authors models.

We have included the suggested analysis within the section "A general model of TOP2B binding based on DNase-seq, RAD21 and CTCF” (lines 512-529). The corresponding results are illustrated in S10 Figure. Our model clearly outperforms the merged DNAseI-CTCF-Rad21 peak set, which identifies numerous false-positive regions with absent experimental TOP2B signal.

2.) It has been known for some time that regions of open chromatin are “sticky”. This has been seen when using IgG controls in ChIP-Seq experiments and when ChIPing proteins which do not associate with chromatin like GFP. This is a aprticulare problem if the washing is not stringent enough. I would like the authors to process an IgG control ChIP-Seq data set for one other their cell lines (at similar read density to the TOPO2 Beta data set), call peaks using similar parameters as

used to call TOPO2 Beta peaks and determine the overlap with their TOPO2 Beta peaks and predicted peaks as seen in Figure 5 B, E. Can the analysis be included in the supplement?

We agree with Reviewer #2 that using IgG can be problematic because of the potential stickiness of open chromatin sites, so we have followed his/her suggestion for all the peak calling analyses performed in the paper, with the exception of the initial one where we identify TOP2B binding sites for training purposes, since our previous results had already validated the utility of this set of peaks for the generation of predictive models. Since the read densities of test and control samples affect peak calling results, this change yielded different peak numbers in Figure 6 (Figure 5 in the previous version). Nonetheless, these modifications have not led to any significant difference in the overall results nor the main findings of our study.

3.) It is important to validate antibodies prior to using them for ChIP-Seq experiments. I don't see that the authors have validated the TOPO2 Beta antibodies used for the ChjIP-Seq experiments. Can a extract of total cellular proteins be resolved on a 4-20% gradient gel and the antibodies used for ChIP-Seq be used to do a Western blot? Is one band observed at the expected molecular weight? Can this be added to the supplement?

We have now validated the antibody used, both in human and mouse cells (S14 Figure) by performing western blot analysis in wild-type as well as TOP2B-depleted and -deleted cells.

4.) I don't see anywhere in the manuscript that the code to run the model is available. I do see a GEO deposit number. Does this have the code to run the model? I am assuming it is not available to the reviewers as a reviewer link and password is not provided. The code needs to be made freely available. Detailed instructions should be provided on how to run the code, using CTCF, RAD21 and DNAseI inputs, to get the predicted TOPO2 Beta output.

The code has been documented and made public in GitLab: https://gitlab.com/mgarciat/genome-wide-prediction-of-topoisomerase-iibeta-binding

Minor Points
1.) Can a key be added to 1B and 1D? as in figure 1C? It is difficult for the reader to go to the figure legend to determine what the groups are.

A key has been added to Figures 1B and 1D.

2.) Can the ChIP-Seq peak calls and the "predicted" peak cells be added for each track in Figure 6 A and B? Also, supplemental Fig S6, S7 and Fig 1A?

As kindly suggested by the reviewer, and since Figure 6A illustrates comparative peak analyses, we have included peak tracks for experimental and predicted TOP2B signals. We prefer not to include them in the other Figures in which peak analysis is not performed, but we are of course open to reconsider.

Reviewer #3

Summary:
Martinez-Garcia et al., built several machine learning models to predict the genome-wide binding of topoisomerase II beta (TOP2B) using sequence features, DNA shape, and chromatin properties obtained from publically available data. The authors showed that open chromatin and occupancy of chromatin architecture proteins are consistently the best predictors. Using only DNase I signal,

CTCF, and RAD21 binding, the authors were able to accurately predict TOP2B binding. With experimental validations, the authors showed that the predictive power of these three features is conserved across cell types and between human and mouse, achieving performance similar to biological replicates. Furthermore the predicted probability of TOP2B binding highly resembles experimental data showing the potential of using such a model to achieve quantitative predictions.

We find the work by Martinez-Garcia et al. to be interesting and the manuscript well-written and should be of general interest to genome biologists and those with specific interests in DNA topoisomerases and DNA repair. This is a computational biology paper takes published observations that TOP2B correlates well with cohesin and DNA and formalizes them into a predictive model. Understanding TOP2B binding has important implications yet experiments are often limited by the availability of antibodies. Thus, the predictive model proposed in the manuscript is a valuable approach that could be applied by others. The authors evaluated different models and feature selection methods. Reasonable approaches appear to be taken both computationally and experimentally. For example, the authors carefully tested and interpreted the usage of GC-corrected background sets and used two different TOP2B antibodies for experimental validation.

Major comments:

The major concerns I have come from:

1) Apparent lack of availability of data (no reviewer token was given and so I could not check the if appropriate data and metadata was submitted. Small but crucial details such as the specific antibodies (e.g. no part number was provided) used were not included in the methods and there was no way to assess whether this, along with other crucial metadata was included in the GEO submission referred to.

Although we provided the GEO accession number in the first version of the manuscript, the corresponding token was missing. The data can now be accessed on GEO GSE141528 using the following token: kvknomqerfirzet. We apologize for the inconvenience.

2) Equally important would be to address the lack of code and intermediate files that would allow others, including reviewers, to replicate the findings. Given this paper uses existing and appropriate models to make useful predictions, it would be most important that other computational biologist can easily replicate them and make new predictions.

The code has been documented and made public in GitLab: https://gitlab.com/mgarciat/genome-wide-prediction-of-topoisomerase-iibeta-binding

3) There seems to be no direct acknowledgement and exploration (aside from a citation) of recent papers that generated original data, and made computational models, to reveal and predict the location of TOP2 covalent complexes. Specifically the original END-seq method has been demonstrated to capture TOP2 covalent complexes (Canela et al. 2017 PMID: 28735753 and PMID: 31202577). In Canela et al. 2017 PMID: 28735753 they presented a linear regression model that could predict DNA breaks (TOP2cc’s) using only RAD21. It would be important for the authors to more directly relate their work to this study. Given this current study and the previous study used MEFs there would be specific opportunities to see how well the author’s current model predicts the END-seq (TOP2cc) data from Canela et al. 2017. At the very least some discussion is warranted giving both studies highlight the importance of cohesin in their predictions.

We have included the suggested analysis within the new section "Prediction of TOP2-induced DSBs” (lines 658-679). The corresponding results are illustrated in S16 Figure. We think this

analysis has substantially contributed to improve our study, and we thank the referee for the insightful suggestion.

Detailed comments:

1) Method: For processing: “raw reads were merged for biological replicates”. For identification of binding sites: “when replicates were possible, peaks were called for individual replicates and overlapping peaks were kept”. It is thus not clear if biological replicates were merged prior to alignment or not.

Peaks from individual biological replicates were overlapped after alignment. To make it clear in the manuscript, we have re-written the corresponding paragraph from “Materials and methods” section (lines 68-74).

2) Method: it is not exactly clear how the additional set of GC-matched controls was generated.

To clarify this question, we have extended our previous explanation from “Materials and methods” (lines 80-88).

3) Method: the features used clearly violates the assumptions of NB. The authors did not clarify why they were still interested to try out the method.

We understand the reviewer’s concerns. However, Naive Bayes (NB) is one of the most efficient and effective inductive learning algorithms for machine learning, achieving a surprisingly good performance in classification even in cases of strong dependences among attributes. This is one of the claims that, for example, Harry Zhang (2004) made when he comprehensively explored which conditions are sufficient and necessary for NB classifiers optimality. According to this study, NB can still be optimal even in cases of strong dependences among attributes.

Zhang, H. (2004). The Optimality of Naive Bayes. In V. Barr & Z. Markov (eds.), FLAIRS Conference (p./pp. 562-567), : AAAI Press. ISBN: 1-57735-201-7.

4) Are the random controls showing similar genomic distribution (distance to TSS for example) as TOP2B peaks?

We have included a peak distribution analysis that is shown in S3 Fig A and B. Accordingly, we have also included a new section within “Materials and methods” called “Distribution of TOP2B binding sites across the genome” (lines 110-124).

5) Line 233-234, 15 experiments used for model training is not clearly indicated in table S1. It is thus not clear which datasets were used for the classification shown in Table 1. This is then explained in lines 272-277. However, it will be more clear if it’s explained before getting into prediction results.

We have included a new column in S1 Table that indicates whether a given dataset has been used for training, test or other analysis.

6) Interpretation: line 297, “These observations contrast with previous findings of TOP2B being involved in transcriptional regulation [47], and may indicate that other chromatin features, such as histone marks and DNase-seq, are capturing such associFation..” Would the authors expect difference performance if they used RNAP2 instead of RNA-seq? Could predictions for TOP2B at specific genomic regions (TSS/first exon) be a more appropriate question when RNA-seq is used?

More details in the discussion how these results contrast previous results would be welcomed. For instance, the association of TOP2B occupancy with cohesin and DNAse I has been revealed in several studies as has its association with transcribed genes. Canela et al.'s END-seq work PMID: 28735753 clearly showed that without transcription a linear model with RAD21 binding could predict TOP2cc genome wide. To me it seems the results here from Martinez-Garcia et al are congruent with previous work. Discussing this would be relevant. Furthermore, the fact that transcription was relevant for TOP2 mediated DSBs is worth mentioning (using modified END-seq from Canela et al. PMID: 31202577 along with Gothe et al (PMID: 31202576)).

We agree with the reviewer. We have now rephrased that section (lines 376-381) to clarify that the fact that Pol2 and RNA-seq datasets prove as poor predictors of TOP2B binding in our analysis does not necessary contradict previous findings, but just reflects that other chromatin features that correlate with transcription are likely capturing this association. We have also specifically mentioned and discussed the described association between TOP2-DSBs and positive transcription reported in Canela et al (2019) and Gothe et al (2019), as suggested.

7) Analysis: Fig1D, GG signal peak seems to be biased on one side? Is this driven by a few regions?

The reviewer is correct there is a bias in GG signal that is not general, but caused by a group of regions, as determined by cluster analysis on GG frequency around TOP2B peaks (see below). However, we think this analysis is out of the context and scope of our findings, and would rather not include it in the manuscript.

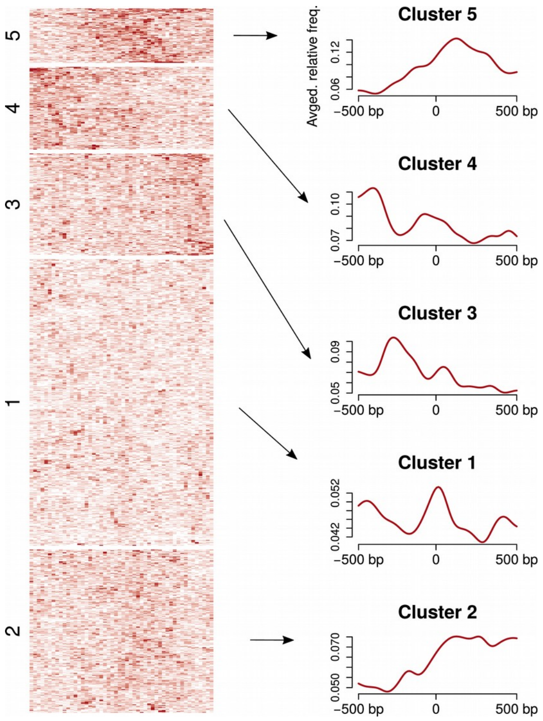

8) Analysis: Fig6, it would be good to add a genome-wide correlation measure between prediction probability and real ChIP-seq data.

We performed Pearson's correlation coefficient analyisis comparing our predictions and the two validation systems (thymus and MCF7), which we have reported in Tables S5 and S6. These tables are cited in lines 564-565 and 619-620.

9) Analysis: The proposed model is trained using TOP2B peaks but the prediction is made on sliding windows across the genome. Could the authors train the model using whole-genome sliding windows or comment on the potential/necessity of doing so. Is it possible to adopt other models to be able to predict the quantitative signal (instead of classification)?

Indeed, other approaches can be adopted for genome wide prediction of quantitative signals. An example is using regression instead of binary classification. This way the whole genome could be used to feed the models instead of positive (TOP2 binding) and negative (random) regions. This is something that we are currently considering to implement in order to predict other sequencing signals, and we thank the reviewer for the suggestion, but is outside the scope of the current manuscript. In any case, we think that one important and surprising point of our analyses is that binary classification can, by computing probability, accurately capture binding intensity. Indeed, we believe this operates by somehow mimicking the nature of the ChIP-seq experiments in which the DNA fragment is either immunoprecipitated or not, and the strength of the signal is a reflection of the probability of this to occur within the entire population of cells.

Reviewer #4

In the manuscript, the authors proposed a computational approach to predict TOP2B binding sites using chromatin accessibility and architectural proteins. The authors compared the performance of three classifiers: Naive Bayes, Support Vector Machine and Random Forests on a bunch of different features including: histone marks, Pol2 binding, architectural components, chromatin accessibility, gene expression, DNA shape, DNA sequence and CpG methylation. Next, they conducted feature selection and found that DNase I hypersensitivity, CTCF and cohesin binding are the most important features to predict TOP2B binding sites. Then, they trained a generalized model using these three features in one cell type and applied the model to a different cell type and validated the predictions with ChIP-seq of TOP2B, showing that the generalized model can predict TOP2B binding sites in new cell line and species.

TOP2B plays important roles in DNA metabolism and 3D organization of chromatin. But currently there are few TOP2B ChIP-seq datasets available. Based on the high accuracy of the predictions presented in the manuscript, this approach offers an attractive way to predict TOP2B binding in different cell types and tissues using the public available datasets. The manuscript is well written and is sound and accurate in general.

Comments

1. Why is Random Forests added after feature selection, not at the beginning of the comparing different features and feature selection? RF can also identify the most important features. Would the important features selected by RF be consistent with the ones selected by FCBF and SS?

We agree with the reviewer, and actually Random Forests (RF) was our first option, but unfortunately could not deal with the dimensionality and the large data volume of the training set. However, our analyses showed that the chromatin features with highest dimensionality, namely DNA sequence and DNA shape, were poorly informative. Therefore, following the reviwers's suggestion, we applied RF to a reduced version of the training data for which only the 15 high throughput sequencing experiments from Table S1 were included. As expected, RF performance was similar to that obtained by naive bayes and support vector machines, and feature importances were quite consistent with our feature selection analyses. The results of this new analyses are included in S7 Figure and S4 Table.

2. In the “Feature selection algorithms” method section, the authors mention that FCBF uses SU as goodness function in line 124, and then mention CFS measure is used as goodness function in line 138. Is CFS used instead of SU in FCBF? Could the authors provide more details?

We agree that the provided description was confusing and we have rephrased the corresponding paragraph (lines 187-188).

3. Could the authors add the comparison of models trained using all features vs three important features to validate that the prediction accuracy is not reduced much.

We have included the suggested analysis in S5 Figure and lines 467-469.

4. For feature selection, SS selects DNase-seq, RAD21 and STAG2 as important features in liver, while DNase-seq, RAD21 and CTCF in MEF. Could the authors compare the performance of the model trained using DNase-seq, RAD21 and CTCF with the model trained using DNase-seq, RAD21 and STAG2 in liver to support that the performance is similar?

We have included the suggested analysis in S6 Figure, S3 Table and lines 474-477.

5. The authors compared the predicted peaks with HOMER detected peaks. Did the authors compare this predictive model with other models that can predict TF binding sites (e.g. DRAF)?

We have encountered some issues when identifying binding sites using popular peak callers due to samples heterogeneity. In order to follow the reviewer’s suggestion, we tried to use DRAF but it seems to be specifically designed to be applied to sequence-dependent transcription factors. According to its website “DRAF utilizes models based on TFBS DNA sequence and TF amino acid properties to provide predictions”. In the corresponding paper, the authors state that they used TFBS sequences from the HOCOMOCO database. After some filtering, they end up with the following 426 TFBS models (401 total TFs): http://autosome.ru/HOCOMOCOS/hocomoco_ad/hocomoco_ad.html. TOP2 is not among such TFs, which indeed make sense since it does not bind to specific motifs.

6. Did the authors apply this approach to predict other TF binding sites, e.g. TOP2A? How is the performance of that?

This is a very interesting point, and we thank the reviewer for the suggestion, but falls outside the scope of the current manuscript. Indeed, we are currently working on the application of our predictive framework to different intermediates of TOP2 dynamics, such as TOP2ccs and TOP2-mediateds DSBs (work in progress), as well as, as suggested by the reviewer, other topoisomerases, such as TOP1 or TOP2A. We consider current manuscript as a proof-of-principle for the genome wide prediction of this type of enzymes that do not show clear dependence on DNA sequence for binding to chromatin.

7. The authors should provide more description about the machine learning framework. It is not clear what package if any was used. Code and scripts should be provided along with the training data. It would be nice to provide the folds of validation too.

The code has been documented and made public in GitLab: https://gitlab.com/mgarciat/genome-wide-prediction-of-topoisomerase-iibeta-binding
