# Supplementary material for: Genome-wide prediction of topoisomerase IIβ binding by architectural factors and chromatin accessibility
Source: PLoS Comput Biol. 2021 Jan 19;17(1):e1007814. doi: 10.1371/journal.pcbi.1007814 (PMC7845959; doi:10.1371/journal.pcbi.1007814)
Supplement: Attachment — Submitted filename: renamed_7ea32.pdf [file pcbi.1007814.s025.pdf]

Dear Editor,

We would like to thank you for the opportunity to submit a revised version of our manuscript. We would also like to express our thanks to the reviewers for their feedback and helpful comments. Please find below our responses to reviewers' comments (note that line numbers refer to the manuscript version with tracked changes):

Reviewer #1

1. how many million reads were each MCF7 replicate?

The following table shows the required mapping statistics:

Sample	Mapped	Unmapped	Total
Replicate 1	36319719 (82.38%)	7769261 (17.62%)	44088980 (100%)
Replicate 2	45731831 (81.74%)	10214896 (18.26%)	55946727 (100%)
IgG	52374205 (71.59%)	20787833 (28.41%)	73162038 (100%)

Note that, as we mention in the Methods section, libraries were subsampled for peak calling so that both the test and the control samples had the same number of reads (in this case, 36319719, corresponding to the least represented library).

2. some typos - "split" not "splitted" in methods and on line 509 "extent" not "extend" on line 479

These typos have been corrected (lines 183, 455, 478 and 564).

Reviewer #2

Major point 1 - The response to this point is very confusing. I think the authors did a good job of giving the reviewer what they wanted, but the result is very confusing. In supp Fig 10 the authors show that 100% of the R2 peaks overlap with the merged peaks, and ~70% with the predicted peaks. If that is the case why would not 70% of the rows in the Merged-only heat map analysis have the high enrichment scores as seen in the Predicted-only heat map analysis? Similarly for the R1? This heat map analysis does not make sense. The Merged-only heat map shows all very low enrichments, but 70% should be as high as those shown in the Predicted-only heat map.

This is a misunderstanding, and we apologize if this was not made clearer before. The peaks selected are specific for either the merged DNAse-seq/RAD21/CTFC set or our predictions. There is therefore no overlap in the regions shown in the heatmaps. As the reviewer points out, it makes no sense to compare two heatmaps in which 70% of the peaks are the same. This is indicated in S10B Figure Legend and the manuscript text (lines 493-496).

Major point 2 - The requested analysis was not done. Maybe I was not completely clear. I wanted the authors to down load a IGG pulldown ChIP-Seq data set and use it in their peak calling algorithm and determine the overlap with their Top2 ChIP-Seq data sets and the predicted data sets. As is shown now it is very difficult to know what the authors did. I don't see any discussion of IGG controls in any of the text, methods. What was actually done?

Again, this is a misunderstanding. Our experimental peaks are already called against our own generated IGG datasets. This will already account for the potential artefacts of the ChIP-seq

technique due to variable “stickyness” or particular chromatin regions. We understand that this is a better approach than just analysing peaks obtained with a published IGG control as suggested by the reviewer. We have clarified this in the new revised manuscript (lines 92 and 211), and apologize for not making this clear in the previous version.

Major point 3 - The validation is wholly inadequate. I requested a 4-20 gradient gel. What is shown is only a band which is about the correct MW for the target (Top2). I would like to see if the antibodies used in the study cross react with other cellular proteins.

We appreciate the reviewer’s concerns regarding the specificity of the antibodies. We now present the entire blot image, so unspecific bands can also be appreciated. Both blots show a major band at the expected size that disappears upon TOP2B knock down or knock out in human and mouse cells, respectively. We therefore have no doubt that both antibodies detect TOP2B. There is an additional band in the mouse samples that might explain the poor quality of the ChIP-seq data with the Novus antibody, which is clearly outperformed by the Santa Cruz antibody and our predictions. Unfortunately, we can’t compare these two antibodies in Western blots, as the Santa Cruz one is discontinued. In any case, we feel that the specificity of the antibodies is not a relevant issue in our computational study, and actually highlights the advantages of using predictions, even over experimental approaches, if the conditions are not optimal.

Major point 4 - this has been addressed adequately.

Minor point 1 - this has been addressed adequately.

Minor point 2 - 6A- Novus is missing the peak calls, Fig 1A is missing all peak calls, S11, S12, S13, S15, S16 are missing peak calls. It would be best for the reader to see these peaks calls, to understand what is being called in each track and see visually the agreement/disagreement between the tracks.

As indicated by the reviewer, we have included the peak calls in all the suggested figures. The reason why Novus is missing the peak calls is that the peak caller did not detect any in the region corresponding to Fig 6A. As a matter of fact, we only detected 2,570 total peaks in this sample, and we show that our predictions clearly outperform the peak calling algorithm in this case.

Reviewer #3

I think the authors addressed my concerns as well as those of the other reviewers.

Reviewer #4

1) Mislabeled Fig S4 as Fig S3 in multiple places: legend of Figure 3, line 363, line 375.

The indicated mislabeling has been corrected (lines 314, 336, 338, 342, 345, 367, 379 and Fig 3 legend).

2) Table 4 and S8 Fig: The cross-validation performance in Liver based on SVM (76.15 ± 9.76%) is lower than the cross-system performance (Liver/MEF 93.11% and Liver/aB cells 98.21%). Is the accuracy of the cross-validation performance in Liver based on SVM correct? In Figure S6, it looks that the cross-validation performance of SVM using RAD21, DNase and CTCF features in Liver is good (AUC 0.95).

We have repeated the analysis and we found that the cross-validation performance in Liver for NB models included in Table 4 and the AUC value of Figure S8 were incorrect. The performance value is 92.57 +- 2.20 and the AUC is 0.94. We have also found that the SVM performance in MEF (applied to MEF data) is 93.89 +- 0.23 instead of 93.62 +- 3.76. These values have been corrected in Table 4 and S8 Fig. Since the rest of the values remained the same after repeating the analysis for the different systems, we can’t find a specific reason for this mistake. We truly appreciate Reviewer’s comment.

3) In line 502-504, besides the Fig 5 examples, provide the Pearson's correlation of predicted TOP2B binding vs experimental signals like Table S5, S6 to validate that similarities are globally significant.

A new supplementary table (Table S5) has been added including the same analysis as that in Tables S5 and S6, which are now named S6 and S7, respectively.
